# Supplementary material for: Effect of Post-Ruminal Urea Supply on Growth Performance of Grazing Nellore Young Bulls at Dry Season
Source: Animals (Basel). 2023 Jan 6;13(2):207. doi: 10.3390/ani13020207 (PMC9854706; doi:10.3390/ani13020207)
Supplement: Supplementary file 1 [file animals-13-00207-s001.zip › animals-2113041-supplementary.pdf]

Table S1. Median and interquartile range of the relative abundance of the genera in Nellore steers influenced by the use of post-ruminal urea in relation to conventional urea during the dry season.

Domain	Phylum	Genera	Treatments			*P*-value
			CONT	PRU	U+PRU	
Bacteria	Actinobacteriota	*Actinomycetospora*	NI[b]	0.000 ± 0.005[ab]	0.001 ± 0.013[a]	0.030
		DNF00809	0.065 ± 0.017[b]	0.089 ± 0.031[a]	0.068 ± 0.390[ab]	0.051
		Ilumatobacteraceae	0.003 ± 0.012[ab]	0.012 ± 0.030[a]	NI[b]	0.035
		Jatrophihabitans	NI[b]	0.000 ± 0.013[ab]	0.000 ± 0.015[a]	0.089
		Kineococcus	NI[b]	NI[b]	0.000 ± 0.001[a]	0.039
		Kineosporia	NI[b]	0.000 ± 0.044[ab]	0.048 ± 0.058[a]	0.091
		Kribbella	NI[b]	0.000 ± 0.008[a]	NI[ab]	0.060
		Uncultured	NI[b]	NI[b]	0.008 ± 0.012[a]	0.003
	Bacteroidota	*Uncultured*	0.412 ± 0.191[a]	0.296 ± 0.123[b]	0.252 ± 0.108[b]	0.013
		U29.B03	0.075 ± 0.045[a]	0.033 ± 0.015[b]	0.034 ± 0.029[b]	0.014
	Bdellovibrionota	*Bdellovibrio*	NI[b]	NI[b]	0.000 ± 0.003[a]	0.037
	Chloroflexi	*Gitt.GS.136*	NI[b]	0.004 ± 0.014[a]	NI[b]	0.058
		Uncultured	NI[b]	NI[b]	0.000 ± 0.005[a]	0.039
	Desulfobacterot	*Desulfuromonas*	NI[b]	0.000 ± 0.002[a]	NI[b]	0.039
		Desulfobulbus	NI[b]	0.000 ± 0.006[a]	NI[b]	0.039
	Elusimicrobiota	*Endomicrobium*	0.005 ± 0.005[a]	0.000 ± 0.005[b]	0.007 ± 0.001[ab]	0.095
	Fibrobacterota	*Fibrobacter*	0.943 ± 0.262[a]	0.631 ± 0.172[b]	0.655 ± 0.201[b]	0.013
	Firmicutes	*Allobaculum*	NI[b]	NI[b]	0.013 ± 0.066[a]	0.040
		Anaerovorax	0.266 ± 0.071[b]	0.335 ± 0.032[a]	0.266 ± 0.055[b]	0.017
		Bacteroides_pectinophilus_group	NI[b]	0.000 ± 0.011[ab]	0.013 ± 0.014[a]	0.049
		Clostridia_vadinBB60_group	0.007 ± 0.017[b]	0.027 ± 0.005[a]	0.021 ± 0.018[ab]	0.054
		Uncultured	0.030 ± 0.012[ab]	0.035 ± 0.011[a]	0.019 ± 0.007[b]	0.036
		Eubacterium	NI[b]	0.003 ± 0.005[a]	NI[ab]	0.070
		Flavonifractor	NI[b]	0.000 ± 0.001[a]	NI[b]	0.076
		Howardella	NI[b]	0.006 ± 0.017[a]	NI[ab]	0.067
		Lachnospiraceae_NK3A20_group	0.921 ± 0.377[ab]	0.834 ± 0.287[a]	0.611 ± 0.207[b]	0.078
		Lachnospiraceae_NK4B4_group	0.021 ± 0.009[ab]	0.009 ± 0.012[b]	0.016 ± 0.008[a]	0.079
		Lachnospiraceae_XPB1014_group	0.893 ± 0.214[ab]	1.002 ± 0.273[a]	0.817 ± 0.237[b]	0.088
		Lysinibacillus	NI[b]	0.013 ± 0.021[a]	0.012 ± 0.016[a]	0.030
		Peptoclostridium	NI[b]	0.007 ± 0.603[ab]	0.536 ± 1.006[a]	0.037

		Peptococcus	NI[b]	NI[b]	0.000 ± 0.015[a]	0.039
		Staphylococcus	NI[b]	NI[ab]	0.004 ± 0.009[a]	0.021
		Weissella	NI[b]	NI[b]	0.004 ± 0.013[a]	0.026
	Myxococcota	*P3OB.42*	0.003 ± 0.003[a]	NI[b]	0.003 ± 0.003[ab]	0.097
	Planctomycetota	*vadinHA49*	NI[ab]	NI[b]	0.000 ± 0.005[a]	0.092
	Proteobacteria	*Allorhizobium.Neorhizobium.Pararhizobium.Rhizobium*	0.012 ± 0.014[b]	0.048 ± 0.036[a]	0.091 ± 0.083[a]	0.001
		1174.901.12	NI[b]	NI[b]	0.000 ± 0.013[a]	0.011
		Belnapia	NI[b]	NI[b]	0.000 ± 0.001[a]	0.039
		Caulobacter	NI[ab]	0.000 ± 0.005[a]	NI[b]	0.092
		Comamonas	0.004 ± 0.010[b]	0.002 ± 0.010[b]	0.012 ± 0.013[a]	0.065
		Devosia	0.000 ± 0.002[b]	0.014 ± 0.043[ab]	0.022 ± 0.035[a]	0.040
		Hyphomicrobium	NI[b]	0.000 ± 0.006[ab]	0.000 ± 0.009[a]	0.070
		Massilia	0.007 ± 0.024[b]	0.010 ± 0.038[b]	0.047 ± 0.083[a]	0.019
		Pseudomonas	0.033 ± 0.153[b]	0.189 ± 0.173[ab]	0.206 ± 0.089[a]	0.055
		Uncultured	NI[b]	NI[ab]	0.000 ± 0.007[a]	0.088
		Uncultured	0.021 ± 0.019[b]	0.023 ± 0.005[a]	0.013 ± 0.014[a]	0.027
		Rubellimicrobium	NI[b]	NI[b]	0.000 ± 0.062[a]	0.039
	Spirochaetota	*Treponema*	1.015 ± 0.285[a]	0.735 ± 0.431[ab]	0.729 ± 0.317[b]	0.061
	Verrucomicrobiota	*ADurb.Bin063.1*	0.000 ± 0.004[a]	NI[ab]	NI[b]	0.091
Archaea	Halobacterota	*Methanomicrobium*	1.521 ± 1.116[b]	2.206 ± 0.412[ab]	2.648 ± 0.909[a]	0.037

NI: not identified. SEM: Standard error means. CONT: conventional urea; PRU: post-ruminal urea; U+PRU: conventional urea+ post-ruminal urea. Values followed by superscript letters indicate statistical differences ($P < 0.05$) based on Kruskal-Wallis test.
